# Supplementary material for: Retrospective analysis of neurofilament-light chain in patients with inflammatory bowel disease – A pilot study
Source: PLoS One. 2026 Jan 29;21(1):e0340182. doi: 10.1371/journal.pone.0340182 (PMC12854413; doi:10.1371/journal.pone.0340182)
Supplement: S1 File — (DOCX) [file pone.0340182.s001.docx]

**Retrospective analysis of neurofilament-light chain in patients with inflammatory bowel disease – a pilot study**

Running title: Serum NfL in patients with IBD

Andreas W. Wolff^1*^, Emily Feneberg^1^, Julius Shakhtour^2^, Katja Steiger^2^, Roland M. Schmid^3^, Bernhard Haller^4^, Nya Reinhardt^1^, Moritz Middelhoff^3^, David Schult-Hannemann^3¶^ , and Paul Lingor^1,5,6¶^

^1^ Clinical Department of Neurology, School of Medicine and Health, Technical University of Munich, Munich, Germany.

^2^ Department of Preclinical Medicine, Institute of Pathology, School of Medicine and Health, Technical University of Munich, School of Medicine and Health, Munich, Germany.

^3^ Department of Internal Medicine II, School of Medicine and Health, Technical University of Munich, Munich, Germany.

^4^ Institute for AI and Informatics in Medicine, School of Medicine and Health, Technical University of Munich, Munich, Germany.

^5^ German Center for Neurodegenerative Diseases (DZNE), Munich, Germany

^6^ Munich Cluster of Systems Neurology (SyNergy), Munich, Germany.

*Corresponding Author

E-mail: [andreas.wolff@tum.de](mailto:andreas.wolff@tum.de)

^¶^These authors contributed equally to this work.

Supplementary Information: Detailed test statistics

Demographics and clinical characteristics of study participants

1. ANOVA (age between groups): F(2, 69) = 0.84, p-value = 0.44
2. Pearson's Chi-squared test (sex between groups): Χ² = 1.13, df = 2, p-value = 0.61
3. ANOVA (BMI between groups): F(2, 69) = 0.93, p-value = 0.40
4. Pearson's Chi-squared test (current Tabacco consume between groups): Χ² = 3.85, df = 4, p-value = 0.43
5. Pearson's Chi-squared test (physical active between groups): Χ² = 7.92, df = 4, p-value = 0.10
6. Pearson's Chi-squared test (country of birth between groups): Χ² = 7.86, df = 8, p-value = 0.45
7. ANOVA (age at IBD diagnosis between groups): F(1, 47) = 0.46, p-value = 0.50
8. ANOVA (disease duration between groups): F(1, 47) = 0.23, p-value = 0.64
9. Pearson's Chi-squared test (immunosuppressive therapy present between groups): Χ² = 41.46, df = 2, p-value < 0.001

Serum NfL and clinical parameters

1. Pearson's product-moment correlation (correlation of age and NfL): r = 0.66, 95% CI [0.50–0.77], t = 7.23, df = 69, p-value <0.001
2. Welch Two Sample t-test (NfL between sexes): t = -0.25, df = 67.93, p-value = 0.81
3. Pearson's product-moment correlation (correlation of BMI and NfL): r = 0.14, 95% CI [-0.10–0.36], t = 1.14, df = 69, p-value = 0.26
4. Type III ANOVA (NfL between groups): F(2, 68) = 0.65, p-value = 0.53.
5. Age-adjusted linear regression model (NfL between groups):
   1. global Type III ANOVA: F(2, 67) = 0.34, p=0.71
   2. age: regression coefficient (β) (95% CI): 0.03 (0.02 – 0.04), p-value < 0.001
   3. group: Comparison to control:
      1. Crohn’s disease: β (95% CI): -0.07 (-0.42 – 0.29), p-value = 0.70
      2. Ulcerative colitis: β (95% CI): -0.14 (-0.50 – 0.21), p- value =0.42
6. Age-matched paired pairwise t-tests (NfL between groups):
   1. Control vs. Crohn’s disease: t = 1.36, df = 22, p-value = 0.19
   2. Control vs. Ulcerative colitis: t = 1.52, df = 22, p-value = 0.14
   3. Crohn’s disease vs. Ulcerative colitis: t = -0.36, df = 23, p-value = 0.72
7. Kruskal-Wallis rank sum test (CRP between groups): Kruskal-Wallis Χ² = 10.99, df = 2, p-value = 0.004
8. Pairwise Wilcoxon rank sum test with continuity correction (CRP between groups):
   1. Control vs. Crohn’s disease: W = 127, p-value = 0.002
   2. Control vs. Ulcerative colitis: W = 176, p-value = 0.027
   3. Crohn’s disease vs. Ulcerative colitis: W = 370.5, p-value = 0.15
9. Pearson’s Chi-square test (macroscopic inflammation during colonoscopy between groups): Χ² = 34.64, df = 2, p-value < 0.001
10. Pearson's product-moment correlation (correlation of age and disease duration): r = 0.65, 95% CI [0.45–0.79], t = 5.83, df = 46, p-value < 0.001
