# Supplementary material for: Retrospective analysis of neurofilament-light chain in patients with inflammatory bowel disease – A pilot study
Source: PLoS One. 2026 Jan 29;21(1):e0340182. doi: 10.1371/journal.pone.0340182 (PMC12854413; doi:10.1371/journal.pone.0340182)
Supplement: Attachment — Submitted filename: Response to reviewers.docx [file pone.0340182.s003.docx]

Dear Editors, dear Reviewer,

Thank you very much for your kind suggestions.

In the following we respond to your comments point by point. Citations of our initial manuscript version are formatted in *italics*. Changes to the original text are given in *italics and underlined*.

**Editor comments:**

**Question 1:** 1. Please ensure that your manuscript meets PLOS ONE's style requirements, including those for file naming. The PLOS ONE style templates can be found at….

**Reply 1:** We changed manuscript style, and it now meets the PLOS ONE’s style requirements.

**Question 2:** 2. Thank you for stating in your Funding Statement:

“KS, MM, and DSH received funding from the German Research Foundation (DFG, CRC1371, Project number 395357507, https://www.dfg.de/en).”

Please provide an amended statement that declares *all* the funding or sources of support (whether external or internal to your organization) received during this study, as detailed online in our guide for authors at http://journals.plos.org/plosone/s/submit-now. Please also include the statement “There was no additional external funding received for this study.” in your updated Funding Statement.

Please include your amended Funding Statement within your cover letter. We will change the online submission form on your behalf.

**Reply 2:** Thank you for this input, we adapted funding statement and cover letter accordingly.

**Question 3:** 3. We note that you have indicated that there are restrictions to data sharing for this study. For studies involving human research participant data or other sensitive data, we encourage authors to share de-identified or anonymized data. However, when data cannot be publicly shared for ethical reasons, we allow authors to make their data sets available upon request. For information on unacceptable data access restrictions, please see http://journals.plos.org/plosone/s/data-availability#loc-unacceptable-data-access-restrictions.

Before we proceed with your manuscript, please address the following prompts:

a) If there are ethical or legal restrictions on sharing a de-identified data set, please explain them in detail (e.g., data contain potentially identifying or sensitive patient information, data are owned by a third-party organization, etc.) and who has imposed them (e.g., a Research Ethics Committee or Institutional Review Board, etc.). Please also provide contact information for a data access committee, ethics committee, or other institutional body to which data requests may be sent.

b) If there are no restrictions, please upload the minimal anonymized data set necessary to replicate your study findings to a stable, public repository and provide us with the relevant URLs, DOIs, or accession numbers. Please see http://www.bmj.com/content/340/bmj.c181.long for guidelines on how to de-identify and prepare clinical data for publication. For a list of recommended repositories, please see https://journals.plos.org/plosone/s/recommended-repositories. You also have the option of uploading the data as Supporting Information files, but we would recommend depositing data directly to a data repository if possible.

Please update your Data Availability statement in the submission form accordingly.

**Reply 3:** We added the following to the Data availability statement: *To protect participant privacy and comply with data protection regulations imposed by the responsible Ethics Committee individual-level data are made available to the public upon request.* *Requests for access to the research data should be directed to the Department of Neurology at TUM University hospital, Technical University of Munich (TUM), Munich, Germany (neurologie@mri.tum.de), where approval will be coordinated with the responsible Ethics Committee at TUM before any data are released.*

**Question 4:** 4. In the online submission form you indicate that your data is not available for proprietary reasons and have provided a contact point for accessing this data. Please note that your current contact point is a co-author on this manuscript. According to our Data Policy, the contact point must not be an author on the manuscript and must be an institutional contact, ideally not an individual. Please revise your data statement to a non-author institutional point of contact, such as a data access or ethics committee, and send this to us via return email. Please also include contact information for the third party organization, and please include the full citation of where the data can be found.

**Reply 4:** Please see Reply 3.

**Question 5:** 5. Thank you for stating the following in your manuscript:

“Katja Steiger, Moritz Middelhoff, and David Schult-Hannemann received funding from the German Research Foundation (DFG, CRC1371, Project number 395357507).”

We note that you have provided additional information within the Acknowledgements Section that is not currently declared in your Funding Statement. Please note that funding information should not appear in the Acknowledgments section or other areas of your manuscript. We will only publish funding information present in the Funding Statement section of the online submission form.

Please remove any funding-related text from the manuscript and let us know how you would like to update your Funding Statement. Currently, your Funding Statement reads as follows:

“KS, MM, and DSH received funding from the German Research Foundation (DFG, CRC1371, Project number 395357507, https://www.dfg.de/en).”

Please include your amended statements within your cover letter; we will change the online submission form on your behalf.

**Reply 5:** We apologize for the confusion and would like to clarify this: The Acknowledgement section currently states: *We thank the tissue biobank of Klinikum rechts der Isar and TUM (IBioTUM) namely, Klaus-Peter Janssen and Anja Conrad, for excellent technical support.*

This refers to the tissue biobank funded through German Research Foundation (DFG, CRC1371, Project number 395357507, <https://www.dfg.de/en>) and is not related to additional external funding. Therefore, we ask to remain with the original statement.

**Question 6:** 6. Your ethics statement should only appear in the Methods section of your manuscript. If your ethics statement is written in any section besides the Methods, please delete it from any other section.

**Reply 6:** We removed the ethics statement from the supplementary statements at the end of the manuscript. It now only appears in the Methods section of the manuscript.

**Question 7:** 7. If the reviewer comments include a recommendation to cite specific previously published works, please review and evaluate these publications to determine whether they are relevant and should be cited. There is no requirement to cite these works unless the editor has indicated otherwise.

**Reply 7:** Not applicable.

**Reviewers' comments:**

Reviewer #1:

**Question 1:** The Introduction is quite elaborate, but the most important information are missing i.e. what is known until now about the role of neurofilament-light chain in inflammatory bowel disease, what is the knowledge gap, and why it this area should be explored. Wnhat this study would add to the current knowledge? Please define clear aim of the study.

**Reply 1:** We thank the reviewer for the important remark. We adapted the introduction:

*Taken together, NfL is considered the most promising biomarker for quantifying clinical and subclinical neurodegeneration independent of individual disease mechanisms. Its high sensitivity allows for the early detection of subtle neurodegenerative changes even before clinical symptoms appear, making it a valuable tool for risk assessment, disease monitoring, and evaluating potential neuroprotective interventions.(26) To date, no diagnostic tool is available to assess chronic neurodegenerative changes in patients with inflammatory bowel diseases. Therefore, measuring NfL in patients with inflammatory bowel disease could be beneficial, as emerging evidence suggests a link between chronic systemic inflammation and an increased risk of neurodegeneration, potentially allowing for early intervention and monitoring in this at-risk population. To our knowledge, the potential role of NfL as a biomarker in inflammatory bowel disease has not yet been explored. (3) In this pilot study, we assessed the utility of serum NfL for detecting signs of chronic neurodegeneration in a well-characterized cohort of patients with inflammatory bowel disease at various disease stages, alongside matched healthy controls. Furthermore, we examined how clinical and paraclinical factors might influence NfL levels.*

**Question 2:** The most significant concern refers to methodology.

The study lacks a calculation of the sample size which implies a question about the power of the study to provide statistically and clinically relevant results.

**Reply 2:** This is an important remark, and we acknowledge this limitation. Our study was conducted using a historical cohort of patients with inflammatory bowel disease and matched controls; furthermore, our study is the first to assess NfL levels in IBD patients, therefore no statistics were available to estimate the sample size. Therefore, an a-priori sample size calculation was not performed. We have addressed this limitation explicitly in the discussion section of the manuscript.

We would like to emphasize that previous studies with comparable sample sizes have successfully detected significant changes in NfL levels, even in relatively small patient cohorts. Since this is the first study evaluating NfL in patients with inflammatory bowel disease, no prior estimates of mean values or standard deviations are available for this population.

To provide context, the largest body of evidence for NfL comes from studies on dementia patients, where a mean serum NfL concentration of 19 ± 12 pg/mL has been reported for patients with Alzheimer’s disease, compared to 10 ± 7 pg/mL in healthy controls (PMID: 36196979). Based on these values, a sample size of approximately 23 subjects per group would be required to detect differences using a two-sided t-test with 80% power and a significance level of 0.05. Therefore, we consider the sample size in our study sufficient to detect clinically meaningful differences, although we agree that validation in larger cohorts is warranted.

**Question 3:** The authors also neglect to provide eligibility criteria for the study and control group.

**Reply 3**: We additionally added information on the selection process for this study to the methods section: *Inclusion criteria were age ≥18 years and patients with gastrointestinal disease or suspicion thereof requiring endoscopic examination (colonoscopy and/or upper gastrointestinal endoscopy) or presenting for routine endoscopic follow-up or screening. Exclusion criteria included inability to consent, contraindications for biopsy (e.g., thrombocytopenia <50,000/µl, coagulation abnormalities), and poor general condition (ECOG >2 or Karnofsky <30%).* […] *Age- and sex-matched subjects were selected randomly from the ColoBAC registry. Absence of neurological comorbidities and availability of serum samples was verified manually.*

**Question 4:** Why one patient from healthy control group was on immunosupressive therapy?

**Reply 4**: Thank you for raising this point. We have clarified this detail in Table 1, now indicating that the immunosuppressive treatment administered to this patient was for a renal condition, not related to inflammatory bowel disease. During colonoscopy, we confirmed the absence of intestinal inflammation in this patient, and a thorough review of the medical records verified that the patient had no neurological disorders or relevant comorbidities expected to confound NfL levels.

**Question 5:** Why you did not performed any neurocognitive test?

**Reply 5:** As this pilot study was based on an existing historical cohort of IBD patients, the original study design did not include assessment of neurocognitive function. Given the considerable time elapsed between the baseline sampling and the current follow-up period, we determined that it would not be appropriate to evaluate neurocognitive function at this stage. However, we thank the reviewer for this important question, and this limitation is specifically addressed in the relevant section of the manuscript.

**Question 6:** Clinical characteristics of patients with IBD is missing (phenotype, location, activity, age at onset, co-morbidities etc.)

**Reply 6:** Thank you for your comment. We have added important additional clinical characteristics to Table 1, which now includes age at initial diagnosis, disease duration, and disease activity. We chose not to include all available clinical data—for example, detailed comorbidities—due to the complexity and heterogeneity of these variables. However, as stated in the methods section, patients and control subjects with neurological comorbidities known to affect NfL levels were excluded from the study to minimize confounding.

**Question 7:** The discussion does not provide explanation of results, nor any significant implications

**Reply 7:** Thank you very much for your remark. In response, we have revised the manuscript to strengthen the discussion regarding the observed slower age-dependent increase in serum NfL levels among patients with Crohn’s disease. We have more explicitly elaborated on potential reasons that might underly this finding.

Moreover, we have placed additional emphasis on the necessity for prospective longitudinal studies that incorporate multi-modal biomarker panels, advanced neuroimaging techniques, and comprehensive cognitive assessments across diverse populations of patients with inflammatory bowel disease. Such studies are essential to unravel the complex processes involved in neuronal injury and neurodegeneration in this patient population.

Beyond revisions in wording, we also added clear implications to guide future research aimed at identifying clinically relevant subgroups of IBD patients who may present with biomarker evidence of neurodegeneration. This stratification could be crucial for earlier detection and potential therapeutic interventions targeting neurological comorbidities in inflammatory bowel disease.

________________________________________

6. PLOS authors have the option to publish the peer review history of their article (what does this mean?). If published, this will include your full peer review and any attached files.

If you choose “no”, your identity will remain anonymous but your review may still be made public.

Do you want your identity to be public for this peer review? For information about this choice, including consent withdrawal, please see our Privacy Policy.

Reviewer #1: No
