# Supplementary material for: Retrospective analysis of neurofilament-light chain in patients with inflammatory bowel disease – A pilot study
Source: PLoS One. 2026 Jan 29;21(1):e0340182. doi: 10.1371/journal.pone.0340182 (PMC12854413; doi:10.1371/journal.pone.0340182)
Supplement: Attachment — Submitted filename: Response to reviewers and editors.docx [file pone.0340182.s004.docx]

Dear Editors, dear Reviewers,

Thank you very much for your kind suggestions.

In the following we respond to your comments point by point. Citations of our initial manuscript version are formatted in *italics*. Changes to the original text are given in *italics and underlined*.

**Response to Reviewer #2:**

**Question 1:**

The authors provided a thorough revision addressing the reviewers' main concerns. Nevertheless, lack of neurocognitive testing and the relatively small sample size without an a priori power calculation is still an issue. While these are acknowledged in the discussion, they limit the strength of conclusions. Yet, the study is overall technically sound, the data support the main conclusion that serum neurofilament-light chain (NfL) levels show age-dependency but no significant difference between inflammatory bowel disease (IBD) patients and controls, although Crohn's disease showed a distinct age-dependent pattern. The conclusions are appropriately cautious given the limitations.

Minor points:

1) I recommend to emphasize the pilot nature of this study clearly and suggest future larger, longitudinal studies with cognitive testing and multimodal biomarkers.

**Reply 1:**

Thank you for this remark. We adapted title, abstract, and conclusion section, accordingly, now emphasizing the pilot nature of this study.

**Question 2:**

2) I suggest adding a Supplementary Information showing other statistical values besides p value such as t and df values for t-test, df, P value for ANOVA, H and df for Kruskal Wallis etc. Violin box plot showing single data points shall be provided and max/min should show the horizontal termination bar (similar to STD error bars, see also https://www.nature.com/articles/s41598-023-29704-8/figures/1). In addition. wildcards should show significance directly in graphs (Fig. 1B).

**Reply 2:**

Thank you for this important remark. We added comprehensive test statistics as supplementary material. Furthermore, we adapted Figure 1B now depicting a violin box plot. We ask the reviewer to not include “significance” in form of p-values in the graph, since three distinct statistical tests were performed to identify group differences.

**Question 3:**

3) The author sequence is a bit unusual. It says “equal contribution” for the two last authors though this is usually done for first authors. Also, the last author is commonly the PI receiving funds which is here not the case. I suggest to clarify this.

**Reply 3:**

This work was conducted in the context of a biobanking study for which Katja Steger, Moritz Middelhoff, and David Schult-Hannemann received funding. This pilot study itself was initiated by the first author, the study received no dedicated funding, and both senior authors—David Schult-Hannemann from the Department of Internal Medicine II and Paul Lingor from the Department of Neurology—jointly supervised the study. We hope that clarifies the ambiguities.

**Response to Reviewer #3:**

**Question 1:**

(1) In my view, the authors have adequately addressed the concerns of the prior reviewer. Overall, the manuscript is well constructed, and the conclusions largely are justified by the experimental data presented. I have two minor comments for the authors for their consideration:

(2) Methods. Data Acquisition and Classification. This section (with regard to histological findings) is confusing and could be structured for clearer readability. The use of the Nancy Histological Index (Line 165) mentioned at the end of this section stated to be ‘assigned for the highest degree of inflammation per case’ requires some explanation. Clarification would be useful as the NHI rating scale was used to determine histological disease activity in the study (Table 2), but with minimal explanation of the rating scale in the legend.

**Reply 1:**

We thank the reviewer for this important comment and have clarified our manuscript accordingly. We added further information about the Nancy index and clarified that, if patients presented with several different grades of histological inflammation, i.e., areas with low inflammatory activity and areas with high inflammatory activity (in one slide or in several biopsies), the NHI was always based on the area with the highest inflammatory activity. This corresponds to routine pathological diagnostics. We have made the following changes, which we hope will clarify any ambiguities:

*Biopsy samples were processed routinely and HE staining was performed according to standard protocols. Experienced pathologists performed histologic evaluation and diagnosis of IBD was made in accordance with valid recommendations [34] together with clinical information. Each biopsy site was assessed separately. Histologically, chronic inflammation was defined by a mononuclear inflammatory infiltrate. Active inflammation was present with an infiltration of neutrophil granulocytes, in the presence of crypt abscesses, or cryptitis. If there were features of both, chronic and active inflammation, the case was classified as active-chronic inflammation. Degree of inflammation was classified as low, moderate, or high grade, based on the density of the immune infiltrate. Likewise, crypt architectural disorder was classified in three grades (low, moderate, or high) based on the extent and severity of crypt atrophy, crypt shortening, and crypt branching. Each histopathological report was systematically evaluated, and the findings of the individual biopsy positions were summarized for each individual case. When multiple different grades of inflammation or crypt architectural disorder were present, for example low or high inflammatory activity and crypt architectural disorder in a patient, the highest grade per case was noted. In addition, The Nancy Index was employed to assess and grade the histological severity of inflammation. The Nancy Index uses a five-level scale from 0 to 4, with 0 indicating no relevant histological disease activity and 1 to 4 representing increasing degrees of inflammation and tissue damage, up to grade 4, which reflects severe activity such as ulcers or erosions. The Nancy Index was always based on the highest degree of inflammation. [35]*

**Question 2:**

(3) Discussion (Line 272) The authors state: ‘However, higher disease activity was not identified as an independent predictor of higher NfL values in the present cohort, presumably due to small sample size’. Instead of just asserting this null result, the authors might adopt a more cautious phrasing by adding wording such as….in addition to limitation in sample size, the absence of an independent association between disease activity and NfL values may also reflect measurement sensitivity, or the complex relationship between systemic inflammation and neuroaxonal injury.

**Reply 2:** Thank you for this remark, we adapted this section to:

*In consideration of the small sample size, the absence of an independent association between disease activity and NfL values may also reflect measurement sensitivity, or the complex relationship between systemic inflammation and neuroaxonal injury.*
